# Global screening of potential *Candida albicans *biofilm-related transcription factors via network comparison

**DOI:** 10.1186/1471-2105-11-53

**Published:** 2010-01-26

**Authors:** Yu-Chao Wang, Chung-Yu Lan, Wen-Ping Hsieh, Luis A Murillo, Nina Agabian, Bor-Sen Chen

**Affiliations:** 1 Laboratory of Control and Systems Biology, Department of Electrical Engineering, National Tsing Hua University, Hsinchu 30013, Taiwan; 2 Institute of Molecular and Cell Biology, National Tsing Hua University, Hsinchu 30013, Taiwan; 3 Department of Life Science, National Tsing Hua University, Hsinchu 30013, Taiwan; 4 Institute of Statistics, National Tsing Hua University, Hsinchu 30013, Taiwan; 5 Department of Cell and Tissue Biology, University of California, San Francisco, CA, USA

## Abstract

**Background:**

*Candida albicans *is a commonly encountered fungal pathogen in humans. The formation of biofilm is a major virulence factor in *C. albicans *pathogenesis and is related to antidrug resistance of this organism. Although many factors affecting biofilm have been analyzed, molecular mechanisms that regulate biofilm formation still await to be elucidated.

**Results:**

In this study, from the gene regulatory network perspective, we developed an efficient computational framework, which integrates different kinds of data from genome-scale analysis, for global screening of potential transcription factors (TFs) controlling *C. albicans *biofilm formation. *S. cerevisiae *information and ortholog data were used to infer the possible TF-gene regulatory associations in *C. albicans*. Based on TF-gene regulatory associations and gene expression profiles, a stochastic dynamic model was employed to reconstruct the gene regulatory networks of *C. albicans *biofilm and planktonic cells. The two networks were then compared and a score of relevance value (RV) was proposed to determine and assign the quantity of correlation of each potential TF with biofilm formation. A total of twenty-three TFs are identified to be related to the biofilm formation; ten of them are previously reported by literature evidences.

**Conclusions:**

The results indicate that the proposed screening method can successfully identify most known biofilm-related TFs and also identify many others that have not been previously reported. Together, this method can be employed as a pre-experiment screening approach that reveals new target genes for further characterization to understand the regulatory mechanisms in biofilm formation, which can serve as the starting point for therapeutic intervention of *C. albicans *infections.

## Background

*Candida albicans*, the most commonly isolated opportunistic human fungal pathogen, can cause skin and mucosal infections as well as life-threatening systemic infections [1,2]. In healthy individuals, *C. albicans *occurs as a dimorphic commensal colonizer of mucosal membranes in the oral cavity, gastrointestinal tract, urogenital mucosa, and vagina. In immunocompromised patients including those undergoing cancer chemotherapy, organ or bone marrow transplantation and those are AIDS sufferers, this organism can become pathogenic, resulting in proliferative growth on mucosal surfaces locally and systemically [3-5]. *Candida *infections, or candidiasis, are difficult to treat and create very serious challenge in medicine. Mortality rates among patients with candidiasis have been increasing and can be as high as 40% to 60%, especially for those who have bloodstream infections (candidemia) [6-8]. Therefore, to understand the molecular mechanisms underlying the pathogenicity of *C. albicans *is imperative for management of such infections.

Biofilm formation plays an important role in the pathogenicity of *C. albicans*. For example, biofilm can serve as reservoirs for the cells to continually seed infection. Moreover, *C. albicans *biofilm cells are much more resistant than free-living planktonic cells to many antifungal agents. As a result, the biofilm-specific property of *C. albicans *cells has prompted recent interests in the study of biofilm structure, physiology, and regulation, and research into the pathogenicity of *Candida *focusing on the prevention and management of biofilm development and antifungal resistance [6,9]. Biofilms are defined as surface-associated communities of cells surrounded by an extracellular matrix and displaying phenotypic features that differ from their planktonic counterparts [10,11]. The development of *C. albicans *biofilm can be divided into four sequential steps. First, the yeast cells adhere to a foreign substrate (host tissue or medical device). Second, the yeast cells proliferate across the substrate surface and pseudohyphae and hyphae begin to develop. Third, the extracellular matrix is produced and the network of pseudohyphae and hyphae cells is embedded within this matrix. Biofilm will then mature into a complex three-dimensional structure. Finally, the progeny biofilm cells disperse to enable remote surfaces to be populated [6,9,10]. Although previous studies have provided some insights, the details of molecular mechanisms that are responsible for biofilm formation still await to be elucidated.

Recently, the *C. albicans *genome for strain SC5314 was sequenced [2], revealing that almost two-thirds of its ~6000 open reading frames are orthologous to genes of *Saccharomyces cerevisiae*, a well-studied model organism and the first eukaryotic organism to have its entire genome sequenced [1,4,12]. In addition, the ease of genetic/molecular manipulation and the development of various tools for genome-wide functional analysis have led to accumulate a large amount of data from the study of *S. cerevisiae*. Since *C. albicans *and *S. cerevisiae *are closely related, i.e., both fall within the hemiascomycete group, the information from *S. cerevisiae *could be adapted and useful for our understanding in *C. albicans *biology and pathogenesis [1,13].

**Figure 1:**
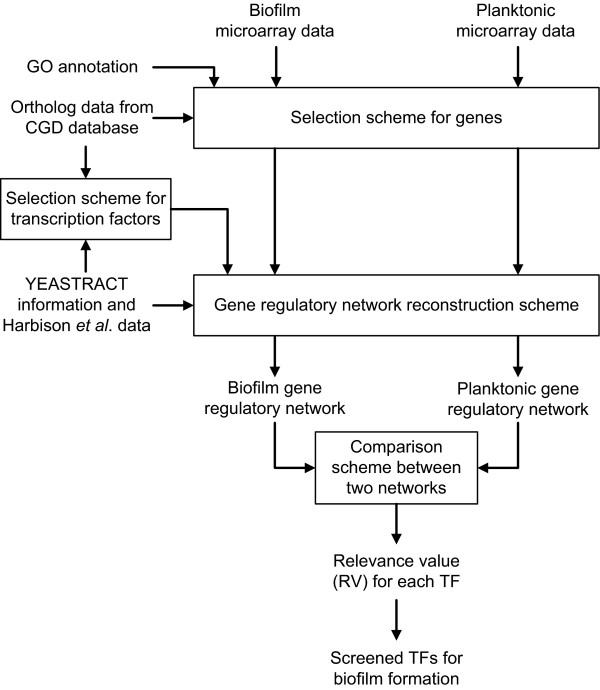
**The flowchart of the proposed method for screening potential biofilm-related TFs in *C. albicans***.

We are investigating the underlying molecular mechanisms that are responsible for biofilm formation in *C. albicans*. Specifically, it is aimed to unravel what makes the difference between biofilm and planktonic cells from the gene regulatory network point of view. Gene regulatory networking is achieved by the action of multiple transcription factors (TFs) binding to *cis*-regulatory DNA elements of the target genes, in response to different environmental signals. Since transcription factors are central to gene regulatory networks, in this study, we developed a computational framework for global screening of potential *C. albicans *biofilm-related TFs via network comparison (Figure 1). We integrated different kinds of data from genome-scale analysis, including gene expression profiles of biofilm formation from *C. albicans *[3], regulatory associations between TFs and genes adopted from *S. cerevisiae *[14,15], ortholog data between *C. albicans *and *S. cerevisiae *genes [16], and Gene Ontology [17]. By using this information, the gene regulatory networks for biofilm cells and planktonic cells were constructed separately. These gene regulatory networks were then compared based on the network structure to reveal their differences and to identify their relevance to biofilm formation for each TF via the so-called 'gain-of-function' and 'loss-of-function' subnetworks. The significance for the potential TFs was determined by statistical analysis. A total of twenty-three TFs are identified to be related to the biofilm formation; ten of them are previously reported by literature evidences. These results indicate that our approach can be useful to reveal TFs significant in biofilm formation and importantly, provide new targets for further studies to understand the regulatory mechanisms in biofilm formation and the fundamental difference between biofilm and planktonic cells.

## Methods

### Overview of the proposed screening method

The method of the global screening for biofilm-related TFs was divided into three key steps: (i) selection scheme for TFs and genes, (ii) scheme for gene regulatory network reconstruction, and (iii) comparison scheme between two networks of biofilm cells and planktonic cells. The output of the method is a score named relevance value (RV) for each TF. RV is computed to correlate the TF with regulation of biofilm formation. A higher score suggests that the particular TF is more likely involved in the regulatory network for *C. albicans *biofilm formation. Based on the RVs, the biofilm-related TFs are chosen. The whole process of the proposed screening method is shown in Figure 1. The data used and the details of each step are described in the following sections.

### Data used in the proposed screening method

In this study, four kinds of data are integrated-microarray gene expression profiles, regulatory associations between TFs and genes, ortholog data between *C. albicans *and *S. cerevisiae *genes, and Gene Ontology annotation information. The microarray data were obtained from Murillo *et al. *[3], in which genome-wide transcription analysis of biofilm formation are profiled using Affymetrix oligonucleotide GeneChips representative of the entire genome of *C. albicans*. Briefly, the DNA microarray includes 7116 ORFs and each microarray experiment was performed in duplicate [3]. The resulting time-course microarray data contain two sets of information for biofilm and planktonic cells, generated from early stages of biofilm formation (0-390 mins, 6 time points). The regulatory associations between TFs and genes were obtained from *S. cerevisiae *using YEASTRACT database http://www.yeastract.com/ and genome-wide location analysis of yeast TFs from Harbison *et al. *[14]. YEASTRACT (**Yea**st **S**earch for **T**ranscriptional **R**egulators **A**nd **C**onsensus **T**racking) deposits more than 34469 regulatory associations between TFs and target genes in *S. cerevisiae*, based on more than 1000 bibliographic references [15]. The genome-wide location analysis allows protein-DNA interactions to be monitored across the entire yeast genome by combing a modified Chromatin Immunoprecipitation (ChIP) procedure with DNA microarray analysis. In Harbison *et al. *[14], the genomic occupancy of 203 DNA-binding TFs in *S. cerevisiae *was determined. The *p*-value threshold for significant binding was selected as *p *≦ 0.001 since their analysis indicated that the threshold maximizes inclusion of legitimate regulator-DNA interactions and minimizes false positives [14]. The ortholog data between *C. albicans *and *S. cerevisiae *genes were retrieved from Candida Genome Database or CGD http://www.candidagenome.org/[16]. Gene orthology and its best hit mappings were used to correlate *S. cerevisiae *genes with *C. albicans *genes using the InParanoid program [18]. The annotations for *C. albicans *genes were acquired from the Gene Ontology (GO) [17]. The GO annotations were facilitated to query for molecular function or biological process of a gene-of-interest in this study. The way we used these data for screening of biofilm-related TFs are further described in the following sections.

### Selection scheme for transcription factors and target genes

**Figure 2:**
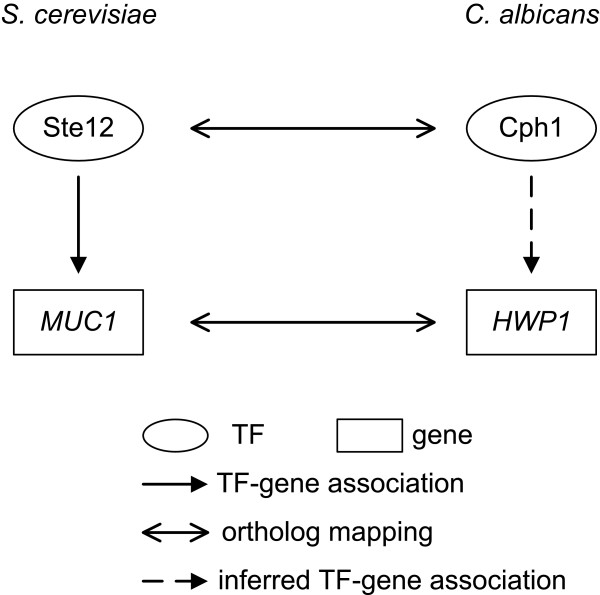
**An example for illustration of *C. albicans *TF-gene regulatory association inference**.

To select TFs and genes for gene regulatory network reconstruction, we included as many TFs as possible in this step. Taking advantages of the fact that *C. albicans *and *S. cerevisiae *are closely related and *S. cerevisiae *is much better characterized than *C. albicans*, the information derived from *S. cerevisiae *was adopted and used in this study. If a *S. cerevisiae *TF has an ortholog found in *C. albicans*, the ortholog was assigned as a TF in *C. albicans*. An example is shown in Figure 2. Ste12 is a well-known transcription factor in *S. cerevisiae *and has a good sequence homologue (named Cph1) in *C. albicans*, this Cph1 protein is thus identified as a TF in *C. albicans*. In this way, TFs are pooled together and will be selected for biofilm-related TFs screening by the proposed method. Notably, some particular *C. albicans *TFs, which have either not been included in the microarray data or lack of association information with target genes, were excluded from the TF pool.

As for the selection of target genes, GO annotations were used [17]. An assumption of the proposed screening method is that if a TF regulates gene expression in biofilm cells rather than in planktonic cells, this particular TF is more likely involved in the regulatory machinery that governs biofilm formation. Therefore, the genes annotated with GO terms such as biofilm formation, or those possibly related to different steps of biofilm formation and development, such as cell adhesion, and filamentous growth, were selected for further analysis. However, if the selected target genes of *C. albicans *are not included in gene expression profiles or have no ortholog mapping data with *S. cerevisiae *genes, they were excluded for the subsequent steps.

The regulatory associations between TFs and genes in *S. cerevisiae *from YEASTRACT database [15] and Harbison *et al. *[14] were used to infer the possible TF-gene regulatory associations in *C. albicans*. An example for this step is illustrated in Figure 2. Borneman *et al. *[19] identified Ste12-*MUC1 *association by chromatin immunoprecipitation (ChIP)-chip experiment with a *p*-value = 2e-15 and the result is deposited in the YEASTRACT database. According to CGD, the TF Ste12 and its target gene *MUC1 *in *S. cerevisiae *have orthologs Cph1 and *HWP1 *in *C. albicans*, respectively. Consequently, based on the experimental results from *S. cerevisiae*, the possible associations between Cph1 and *HWP1 *in *C. albicans *were inferred in our study.

### Gene regulatory network reconstruction scheme

From the first step described above, we have selected TFs, their potential target genes, and their possible regulatory associations. This information was used to further constitute the candidate gene regulatory network [Additional file 1]. A stochastic dynamic model was then applied to prune the candidate network to obtain the gene regulatory networks independently for biofilm cells and planktonic cells, according to their respective data sets. For a target gene *i *in the candidate gene regulatory network, the gene was described using the stochastic discrete dynamic equation (1) [20].(1)

where *x*_*i*_[*t*] represents the gene expression level at time *t *for the particular gene *i*, *a*_*ij *_denotes the regulatory ability of the *j*-th TF toward the *i*-th target gene with a positive sign indicating gene activation and a negative sign indicating gene repression, *z*_*j*_[*t*] represents the regulation function of the *j*-th TF (the *N*_*i *_TFs binding to the target gene *i *are retrieved from the candidate gene regulatory network in Additional file 1), *λ*_*i *_indicates the degradation effect of the present time *t *on the next time *t*+1, *k*_*i *_represents the basal level of expression, *ε*_*i*_[*t*] denotes the stochastic noise due to the model uncertainty and the fluctuation of the DNA microarray data. It has been shown that TF binding usually affects gene expression in a nonlinear fashion, that is, below some level of protein concentration a TF has no effect, while above a certain expression level the effect of the TF may become saturated [21,22]. Thus, the regulation function *z*_*j*_[*t*] was modeled as the sigmoid function, which is one kind of Hill function, of *y*_*j*_[*t*] (the protein concentration profiles of TF *j*) shown in equation (2) [20,22-24].(2)

where *f*_*j *_denotes the sigmoid function, *μ*_*j *_and *σ*_*j *_represent the mean and standard deviation of protein concentration level of TF *j*. The biological implication of the equation (1) is that the gene expression of the target gene *i *at the next time *t*+1 is determined by the present gene expression, the present regulation function of *N*_*i *_TFs binding to this target gene, the degradation effect of the present time, the basal level of gene expression, and some stochastic noises. For each target gene selected from the previous scheme, a stochastic dynamic model was constructed. Consequently, the stochastic dynamic equations for all the target genes constituted the mathematical model of the candidate gene regulatory network.

After constructing the stochastic dynamic model of the candidate gene regulatory network, the microarray gene expression profiles were then overlaid to identify the regulatory parameters in equation (1). Since the DNA microarray data for gene expression profiles of biofilm and planktonic cells are collected separately, the gene regulatory networks of biofilm and planktonic cells can be independently reconstructed. The identification of the gene regulatory network was performed gene by gene, so that the process was not limited by the number of target genes. Due to the non-negativity of basal level of expression (*k*_*i *_≥ 0 in equation (1)), the constrained least squares regression method was used to identify the regulatory parameters [25,26] (see Additional file 2 for details). Moreover, since there are no good data available for genome-wide protein concentration levels in *C. albicans*, gene expression profiles were used instead for identifying the regulatory parameters. Once the regulatory parameters were identified, the significant TF-gene interactions were determined based on the identified *a*_*ij*_'s. By means of Akaike Information Criterion (AIC) [27,28] and student's t-test [29,30], we determined the statistical significance of the interactions between TFs and genes, pruned the candidate gene regulatory network and reconstructed the gene regulatory networks for biofilm and planktonic cells (see Additional files 2, 3, 4 and 5 for details). The resulting biofilm and planktonic gene regulatory networks and the significant TF-gene interactions among them were then used for comparison scheme.

### Comparison scheme between two networks of biofilm and planktonic cells

**Figure 3:**
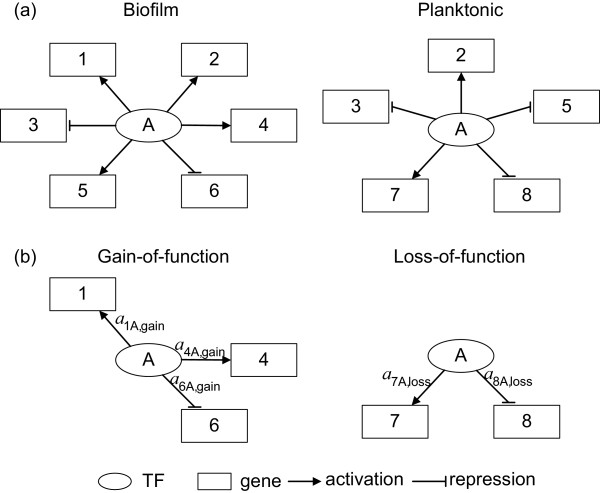
**Schematic diagrams for illustration of gene regulatory network comparison**. (a) The biofilm gene regulatory network and the planktonic gene regulatory network. The regulatory abilities for TF-gene interactions are omitted for simplicity. (b) The gain-of-function and loss-of-function subnetworks after network structure comparison. *a*_*ij*,gain _and *a*_*ij*,loss _indicate the regulatory abilities of the *j*-th TF to the *i*-th target gene for gain-of-function and loss-of-function, respectively, in which a positive sign indicates activation and a negative sign indicates repression.

After the TF/target gene selection and gene regulatory network reconstruction, the gene regulatory networks of both biofilm and planktonic cells and their significant TF-gene interactions were obtained. This information allowed us to compare the networks of biofilm and planktonic cells, and compute the relevance value (RV) to identify TFs that are important in the regulation of biofilm formation. Regardless of the nature of each TF as an activator or a repressor toward its target genes in the gene regulatory networks, we compared the network structure between these two networks. The interactions between TFs and genes in these two networks were simplified as binary relation, in which '1' represents a significant interaction between the TF and the target gene (no matter activation or repression) and '0' denotes no significant interaction (see Figure 3(a) and Table 1 for illustration). As results, comparison of the biofilm with the planktonic gene regulatory network can generate two different subnetworks, one is called 'gain-of-function' subnetwork and the other is 'loss-of-function' subnetwork. If a significant interaction is detected in the biofilm but is absent in the planktonic gene regulatory network, such an interaction is classified into the gain-of-function subnetwork, which represents a subnetwork within the biofilm gene regulatory network. In contrast, if a significant interaction is detected in the planktonic but not in the biofilm gene regulatory network, this interaction is a part of the loss-of-function subnetwork, representing a subnetwork of the planktonic gene regulatory network [Additional files 6, 7 and 8]. Schematic diagrams and the corresponding binary description of TF-gene interactions for elucidation of gene regulatory network comparison are shown in Figure 3 and Table 1.

**Table 1:** Construction of gain-of-function and loss-of-function subnetworks

TF A	Biofilm	Planktonic	Gain-of-function	Loss-of-function
Gene 1	1	0	1	0
Gene 2	1	1	0	0
Gene 3	1	1	0	0
Gene 4	1	0	1	0
Gene 5	1	1	0	0
Gene 6	1	0	1	0
Gene 7	0	1	0	1
Gene 8	0	1	0	1

The table demonstrates the gain-of-function and loss-of-function subnetworks construction shown in Figure 3. The gain-of-function and loss-of-function subnetworks were constructed by comparing the network structure of biofilm gene regulatory network with that of planktonic gene regulatory network via the comparison scheme.

Using the gain-of-function and loss-of-function subnetworks to distinguish the biofilm from planktonic gene regulatory network, we determined a score named relevance value (RV) to quantify the correlation of each TF in these subnetworks with the regulation of biofilm formation, and to identify potential *C. albicans *biofilm-related TFs. To determine the RV for each TF, two important issues are also taken into consideration. First, the magnitude of regulatory abilities *a*_*ij*_'s identified from the gene regulatory network reconstruction scheme denotes the significance of the TF in the transcriptional regulation for a specific target gene. Second, an assumption is made: if a TF regulates more biofilm-related genes in the gain-of-function and loss-of-function subnetworks, then the TF is more likely involved in the regulation for biofilm formation. Consequently, the RV was determined using the following equation, based on the regulatory abilities of TF in the gain-of-function and loss-of-function subnetworks.(3)

where RV_*q *_denotes the relevance value for TF *q*, *a*_*pq*,gain _and *a*_*pq*,loss_, which are numerically obtained from the gene regulatory network reconstruction scheme, indicate the regulatory ability of TF *q *to control the target gene *p *in the gain-of-function subnetwork and loss-of-function subnetwork, respectively; *N*_*q *_and *M*_*q *_represent the numbers of target genes for the TF *q *identified from the gain-of-function and loss-of-function subnetworks, respectively. The implication of equation (3) is that RV quantifies the extent of the TF involved in the interactions with target genes that differentiate biofilm and planktonic gene regulatory networks. The measurement of RV is conceptually similar to the well-known 'graph edit distance' previously used to compare pathways structurally [31]. In the illustrated schematic diagram in Figure 3, the relevance value for TF A is calculated as:(4)

**Figure 4:**
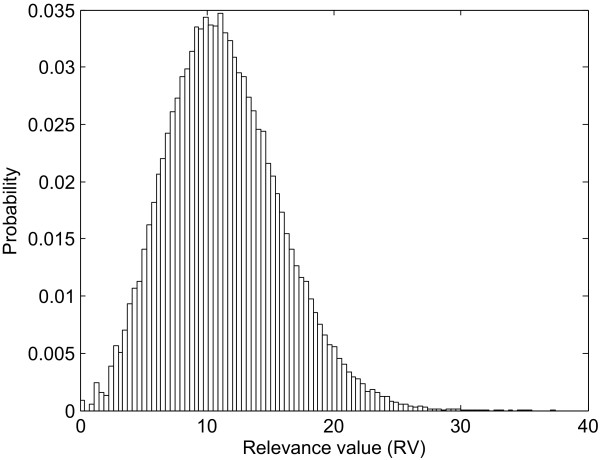
**Distribution of relevance values (RVs) of random network**.

For each TF, a corresponding RV was assigned and an empirical *p*-value was computed to determine the significance of the RV. To determine the *p*-value for an observed RV, a null distribution of RVs (Figure 4) was generated by repeatedly permuting the network structure of the candidate gene regulatory network and computing the RV for each random network structure. The permutation of the network structure was performed by keeping the network size, i.e., the target genes to which a particular TF associated were permuted without changing the total number of TF-gene regulatory associations of the network. Specifically, suppose there are *A *selected TFs, *B *target genes, and *C *TF-gene regulatory associations in the candidate gene regulatory network, the probability of a rewiring of a TF-gene association in the permuted random network is uniformly given by *C*/*AB*. We repeated this process 100000 times and estimated the *p*-value for the corresponding RV as the fraction of random network structures whose RV is at least as large as the RV of the real network structure. The *p*-values were then adjusted by Bonferroni correction to avoid multiple testing problem [29,30]. The RVs with adjusted *p*-value ≦ 0.05 were determined as significant RVs and the corresponding TFs were identified as the potential *C. albicans *biofilm-related TFs.

## Results

### Screening of potential *C. albicans *biofilm-related TFs

We applied the proposed method to analyze and compare data derived from *C. albicans *biofilm and planktonic cells for screening of potential *C. albicans *biofilm-related TFs. Among all *C. albicans *genes, 361 were selected as target genes since they are annotated by at least one of the GO terms, including biofilm formation, cell adhesion, and filamentous growth. By *S. cerevisiae *TF information and the orthologs between *C. albicans *and *S. cerevisiae*, we identified 220 *C. albicans *TFs which have expression profiles in the experiments comparing biofilm with planktonic cells. From the identified TFs and target genes, we further reconstructed the gene regulatory networks for biofilm and planktonic cells, in which 2149 and 2211 TF-gene interactions are included, respectively [Additional files 3, 4 and 5]. Among these two networks, excluding the 1442 common interactions, there are 707 interactions in gain-of-function subnetwork and 769 interactions in loss-of-function subnetwork [Additional files 6, 7 and 8]. We then used the regulatory abilities of TFs in the gain-of-function and loss-of-function subnetworks to compute the RVs for each TF and to determine the significance of these RVs. Consequently, 23 potential TFs related to *C. albicans *biofilm formation were identified and shown in Table 2.

**Table 2:** Identification of potential *C. albicans *biofilm-related TFs

Systematic name	TF*	RV	Adjusted *p*-value^§^	Literature evidence
orf19.5953	orf19.5953	117.6815	< 1e-05	
orf19.610	Efg1	76.1153	< 1e-05	[32,34]
orf19.4433	Cph1	75.4189	< 1e-05	[36]
orf19.5498	Efh1	70.8097	< 1e-05	[39]
orf19.861	orf19.861	68.2492	< 1e-05	
orf19.1773	Rap1	59.4340	< 1e-05	[40]
orf19.837.1	Ino4	53.3481	< 1e-05	
orf19.1069	Rpn4	52.8505	< 1e-05	
orf19.2236	orf19.2236	51.4651	< 1e-05	
orf19.5908	Tec1	51.2743	< 1e-05	[41,42]
orf19.4545	Swi4	49.9914	< 1e-05	
orf19.5041	orf19.5041	45.9961	< 1e-05	
orf19.2054	Fgr15	45.6984	< 1e-05	[43]
orf19.5312	orf19.5312	44.3625	< 1e-05	
orf19.1358	Gcn4	38.4428	< 1e-05	[45]
orf19.7046	Met28	38.4261	< 1e-05	
orf19.4573	Zcf26	35.9090	0.0022	
orf19.971	Skn7	35.7618	0.0022	[46]
orf19.6121	Mnl1	35.4126	0.0022	
orf19.7025	Mcm1	34.7227	0.0066	[47]
orf19.952	orf19.952	32.3603	0.0242	
orf19.5975	orf19.5975	31.8614	0.0286	
orf19.2752	Adr1	31.8191	0.0286	[43]

*The TF names are retrieved from CGD database http://www.candidagenome.org/.

^§^The adjusted *p*-values are obtained by Bonferroni correction.

### The potential biofilm-related TFs

A total of 23 TFs were determined as potential *C. albicans *biofilm-related TFs (Table 2). To assure the effectiveness of our proposed screening method, we seek evidences from literature to validate the inferred functions in regulation of biofilm formation.

(1) Efg1, Cph1 and Efh1: Both cell adhesion and morphogenesis to form hyphae play important roles in biofilm formation and maturation [10]. Efg1 is a downstream transcription factor of Ras-protein kinase A signaling pathway and governs multiple different morphogenetic processes including phenotypic switching and filamentous growth [32-34]. Deletion of *C. albicans EFG1 *gene decreases the ability of the cell to adhere to oral epithelial cells *in vitro *[32].

*C. albicans *Cph1 is an ortholog of *S. cerevisiae *Ste12. In *S. cerevisiae*, the cells mate by responding to pheromones via the functions of mitogen-activated protein kinase (MAPK) cascade and its downstream TF, Ste12. *C. albicans *Cph1 is not only required for mating [35], but is also important for hyphal formation [36]. Finally, *efg1*/*efg1 cph1*/*cph1 *double mutant cannot form hyphae and is also defective in biofilm formation [37,38].

APSES proteins regulate fungal filamentation and differentiation. There are two APSES proteins in *C. albicans*, Efg1 and Efh1 [39]. *C. albicans EFH1 *gene deletion causes hyperfilamentation in an *efg1 *background under certain conditions, indicating that Efh1 modulates and supports the regulatory functions of Efg1 [39].

(2) Rap1 and Tec1: Rap1 is a transcription factor and telomere binding protein that is essential for cell viability in *S. cerevisiae*. Studies from *C. albicans RAP1*-deletion mutant shows that Rap1 is required for efficient repression of pseudohyphal growth under yeast-favoring conditions but is not essential for viability of *C. albicans *[40].

Tec1, a member of the TEA/ATTS family of transcription factors, is shown to regulate hyphal development and virulence in *C. albicans*. Insertion mutations of *TEC1 *cause severe defects in biofilm formation [41,42].

(3) Fgr15, Gcn4, Skn7, Mcm1 and Adr1: Fgr15 is a putative transcription factor with zinc finger DNA-binding motif. Transposon mutation of *FGR15 *affects filamentous growth [43]. Gcn4, like its ortholog in *S. cerevisiae*, activates the transcription of amino acid biosynthetic genes. In addition, *C. albicans *Gcn4 interacts with the Ras-cAMP pathway to promote filamentous growth in response to amino acid starvation [44]. *C. albicans GCN4*-deletion mutant reduces biofilm biomass, indicating that Gcn4 is required for normal biofilm growth [45].

Skn7, one of the response regulator proteins in *C. albicans*, is required for morphogenesis under some conditions and its mutant produces smooth colonies [46]. It is also required for adaptation under some types of oxidative stress *in vitro *[46]. Mcm1 is an essential gene in *C. albicans *whose protein levels are crucial for determination of cell morphology. It might be a mediator recruiting regulatory factors required for hyphal development in *C. albicans *[47]. Adr1, like Fgr15, is also a putative transcription factor with zinc finger DNA-binding motif and its mutant results in less filamentous growth [43].

(4) Other TFs identified: Of the 23 TFs indentified, as described above, 10 of them have been shown to relate to various processes of biofilm formation (e.g. filamentation and cell adhesion) or biofilm formation *per se*. Therefore, the remaining 13 TFs provide good candidates for further experiments to determine their regulatory roles in biofilm formation.

### Statistical measurements of the performance

Among total 220 TFs selected for screening, 23 potential biofilm-related TFs with significant RVs were identified (Table 2). Of the other 197 TFs, we also check literature evidences to see if they are validated by experiments as biofilm-related TFs. Twenty-six out of 197 TFs which do not have significant RV were annotated with GO terms such as biofilm formation, cell adhesion, or filamentous growth. The sensitivity, specificity, positive predictive value, and negative predictive value of the proposed screening method were evaluated (see Additional file 2 for details). The proposed approach can identify potential *C. albicans *biofilm-related TFs with a low sensitivity of 27.78% and a high specificity of 92.93%. Moreover, our method is effective on determining the TFs that are not biofilm-related as the negative predictive value is 86.80%. The positive predictive value is 43.48%, enriching by 2.7-fold the likelihood of screening TFs that are biofilm-related since the biofilm-related prevalence among total 220 TFs is 16.36%. It is noteworthy that these statistics are evaluated based on the published literature evidences and GO annotations, suggesting that if more *C. albicans *biofilm-related TFs are validated by experiments, the statistics should be improved.

## Discussion

The architecture of *C. albicans *biofilms and the correlation between biofilm and infection have been analyzed, but our understanding of the gene regulations that are responsible for the biofilm formation is still limited. Since transcription factors play an important role in gene regulatory networks, here, we develop a computational framework via network comparison to screen for *C. albicans *TFs that may be important for biofilm formation. The original idea is derived from the concept of comparative biology which commonly utilizes comparative approaches in the analysis of genomic sequences to reveal the functional similarities and differences among different species [48]. We extend the concept and compare the gene regulatory networks to explore what makes the difference between biofilm and planktonic cells in *C. albicans*. The advantage of the proposed screening method lies in the convenience and systematicity. Compared with the time- and labor-consuming experiments, we provide an efficient and rapid way for screening TFs by comparing two gene regulatory networks from the systematic point of view. Richard *et al. *[9] used a collection of insertion mutations in 197 *C. albicans *ORFs to screen those mutants that are defective in biofilm formation; however, only 4 such genes are identified. In this study, our computational method has a positive predictive value of 43.48% which is much higher than that shown by Richard *et al. *(~2.03%). Consequently, the proposed screening method can be useful for providing potential target genes for biologists to perform further experiments. It can be considered as a pre-experiment screening. In addition, our approach is not only capable of studying biofilm and planktonic cells, but can also be used to compare two physiological conditions as long as the adequate data are available. For example, this method can be used to screen TFs possibly involved in the cancer development process by comparing the normal cell and cancer cell and the TFs screened could serve as a starting point for therapeutic intervention [49].

Although our approach is shown to be useful, some drawbacks or improvements are still need to be taken in consideration. One assumption of the stochastic dynamic model in equation (1) is that the time delay of transcriptional regulation of the TF to the target gene is only one time unit (about seven minutes in this study), which is not always the case. Previous studies have shown from gene expression profiles that different time delays are required for different TFs to exert regulatory effects on their target genes [21,50,51]. However, since the time delays cannot be experimentally measurable for all the TFs and its potential target genes and the computationally predicted time delays are not completely reliable, the time delays are all set to one time unit when reconstructing the gene regulatory networks. In addition to the time-delay assumption, one important consideration is data accuracy from public domains. For example, based on the orthology information between *C. albicans *and *S. cerevisiae*, we adopt the information of regulatory associations between TFs and genes from *S. cerevisiae *to the study of *C. albicans*. The orthology mappings were performed at CGD using InParanoid software, which basically employs the computed sequence similarity to determine orthologs [18]. If the orthology mapping data is not perfectly accurate, it can result in the misinterpretation of regulatory associations between TFs and genes in *C. albicans*. To overcome the problem, it is better to acquire the TF-gene regulatory associations directly from the experiments (e.g. genome-wide ChIP-chip) using *C. albicans*. Recently, genome-wide location analysis by ChIP (chromatin immunoprecipitation)-chip has been developed for the study of *C. albicans *[52,53]. However, similar studies for biofilm-related TFs are still not available. Another shortage of the information from public domains is the lack of information related to *S. cerevisiae *TF-gene association in YEASTRACT and ChIP-chip data from Harbison *et al. *[14], although orthologs of the TF and target genes do exist in *C. albicans*. Consequently, it will not be able to reconstruct the corresponding gene regulatory network, thus the particular TF is being excluded from the TF pool. One can also solve this problem by performing *C. albicans *ChIP-chip experiments. Once the reliable *C. albicans *TF-gene regulatory associations are obtained, the performance of the proposed screening method can be improved and the reliable gene regulatory networks can be reconstructed.

Numerous factors can affect *C. albicans *biofilm formation, including supporting substrate, growth medium, and *C. albicans *strains [6,9]. Given the complex conditions that affect the kinetics of biofilm formation process and the huge amounts of data generated by post-genomic approaches under different experimental conditions, we can now investigate the most significant TFs that are responsible for the biofilm formation. The screening of biofilm-related TFs is the initial step to elucidate the whole gene regulatory network that governs biofilm formation. Lu and Collins [54] have successfully demonstrated that synthetic biology techniques are feasible to engineer bacteriophage to express DspB, an enzyme that hydrolyzes the crucial biofilm formation adhesin (β-1,6-*N*-acetyl-D-glucosamine) encoded by genes pgaABCD in *E. coli *[55,56], therefore reducing bacterial biofilms. As a result, by combining the systems biology approaches to gain more insight into the molecular mechanisms for biofilm formation with the synthetic biology techniques to engineer the enzyme needed, we may develop new therapeutic strategies to combat the recalcitrant infections caused by *C. albicans *and other microbial pathogens.

## Conclusions

Biofilm formation is a major virulence factor in *C. albicans *pathogenesis and is related to antidrug resistance of this organism. However, little is known about the molecular mechanisms that regulate biofilm formation. In this study, we developed an efficient computational framework for global screening of potential transcription factors controlling *C. albicans *biofilm formation. *S. cerevisiae *information was used to infer the possible TF-gene regulatory associations in *C. albicans*. Gene regulatory networks of *C. albicans *biofilm and planktonic cells were compared to identify the transcription factors involved in biofilm formation and maintenance. A total of twenty-three TFs are identified; ten of them are previously reported to be involved in biofilm formation. Literature evidences indicate that our approach can be useful to reveal TFs significant in biofilm formation and importantly, provide new targets for further studies to understand the regulatory mechanisms in biofilm formation and the fundamental difference between biofilm and planktonic cells, which can serve as the starting point for therapeutic intervention of *C. albicans *infections.

## Authors' contributions

YCW developed the method, performed the analysis, evaluated the results and wrote the manuscript. CYL evaluated the results and revised the manuscript. WPH participated in the statistical analysis and revised the manuscript. LAM and NA provided essential guidance. BSC provided essential guidance and revised the manuscript. All authors read and approved the final manuscript.

## Supplementary Material

Additional file 1 **Supplementary Table S1**. Supplementary table S1 lists the TF-gene regulatory associations in the candidate gene regulatory network. Click here for file

Additional file 2 **Supplementary Methods**. Click here for file

Additional file 3 **Supplementary Table S2**. Supplementary table S2 comprises the significant TF-gene regulatory associations in the biofilm and planktonic gene regulatory networks, respectively. Click here for file

Additional file 4 **Supplementary Figure S1**. Supplementary figure S1 shows the schematic view of the biofilm regulatory network. Click here for file

Additional file 5 **Supplementary Figure S2**. Supplementary figure S2 displays the schematic view of the planktonic regulatory network. Click here for file

Additional file 6 **Supplementary Table S3**. Supplementary table S3 consists of the TF-gene regulatory associations in the gain-of-function and loss-of-function subnetworks, respectively. Click here for file

Additional file 7 **Supplementary Figure S3**. Supplementary figure S3 illustrates the schematic view of the gain-of-function subnetwork. Click here for file

Additional file 8 **Supplementary Figure S4**. Supplementary figure S4 demonstrates the schematic view of the loss-of-function subnetwork. Click here for file
