# Supplementary material for: Global screening of potential Candida albicans biofilm-related transcription factors via network comparison
Source: BMC Bioinformatics. 2010 Jan 26;11:53. doi: 10.1186/1471-2105-11-53 (PMC2842261; doi:10.1186/1471-2105-11-53)
Supplement: Additional file 2 — Supplementary Methods. [file 1471-2105-11-53-S2.PDF]

Supplementary Methods for

Global screening of potential *Candida albicans* biofilm-related transcription factors via network comparison

Identification of the regulatory parameters

After constructing the stochastic dynamic model of the candidate gene regulatory network, the regulatory parameters in the model were needed to be identified using the data we have. The microarray gene expression profiles were then overlaid to identify the regulatory parameters. The identification of the gene regulatory network was performed gene by gene. Before the identification method was determined, the dynamic model must be examined carefully. In equation (1), the basal expression level k_i should be always non-negative, since the microarray expression of the genes are always non-negative. Due to the constraint of the parameters in equation (1), the regulatory parameters were identified by solving the constrained least squares problems.

Equation (1) can be rewritten as the following regression form.

$$\begin{aligned} x_i[t+1] &= \begin{bmatrix} z_1[t] & \cdots & z_{N_i}[t] & x_i[t] & 1 \end{bmatrix} \cdot \begin{bmatrix} a_{i1} \\ \vdots \\ a_{iN_i} \\ (1-\lambda_i) \\ k_i \end{bmatrix} + \varepsilon_i[t] \\ &\equiv \phi_i[t] \cdot \theta_i + \varepsilon_i[t] \end{aligned} \tag{S1}$$

where $\phi_i[t]$ denotes the regression vector which can be obtained from the processing above. θ_i is the parameter vector of the target gene i to be estimated. In order to avoid overfitting, the cubic spline method [1-3] was used to interpolate extra time points for the gene expression data. By the cubic spline method, the values of

$\{z_j[t_l] \quad x_i[t_l]\}$ for $l \in \{1,\ 2,\ \cdots, L\}$ and $j \in \{1,\ 2,\ \cdots, N_i\}$ were easily obtained, where L is the number of expression time points of a target gene *i*, and N_i is the number of TFs binding to the target gene *i*. Equation (S1) at different time points can be arranged as follows

$$\begin{bmatrix} x_i[t_2] \\ x_i[t_3] \\ \vdots \\ x_i[t_L] \end{bmatrix} = \begin{bmatrix} \phi_i[t_1] \\ \phi_i[t_2] \\ \vdots \\ \phi_i[t_{L-1}] \end{bmatrix} \cdot \theta_i + \begin{bmatrix} \varepsilon_i[t_1] \\ \varepsilon_i[t_2] \\ \vdots \\ \varepsilon_i[t_{L-1}] \end{bmatrix} \tag{S2}$$

For simplicity, the notations X_i, Φ_i, and E_i were defined to represent equation (S2) as follows

$$X_i = \Phi_i \cdot \theta_i + \mathrm{E}_i \tag{S3}$$

The constrained least squares parameter estimation problem was formulated as follows

$$\min_{\theta_i} \frac{1}{2} \left\| \Phi_i \theta_i - X_i \right\|_2^2 \quad \text{such that } A\theta_i \le b \tag{S4}$$

where $A = [0 \ \cdots \ 0 \ \ 0 \ \ -1]$, $b = 0$ gave the constraints to force the basal level k_i in equation (1) to be always non-negative, i.e., $k_i \ge 0$. The constrained least squares problem can be solved using the active set method for quadratic programming [4, 5]. Since there are no good data for genome-wide protein concentration levels in *C. albicans*, gene expression profiles were used instead for identifying the regulatory parameters.

Determination of significant interactions

When the regulatory parameters were identified, Akaike Information Criterion (AIC) [6, 7] and student's t-test [8, 9], which is used to calculate the *p*-values of the

regulatory abilities, were employed for both model selection and determination of significant interactions in the gene regulatory network. The AIC, which attempts to include both the estimated residual variance and model complexity in one statistics, decreases as the residual variance decreases and increases as the number of parameters increases. As the expected residual variance decreases with increasing parameter numbers for nonadequate model complexities, there should be a minimum around the correct parameter number [6, 7]. Therefore, AIC can be used to select model structure based on the regulatory abilities (a_{ij}'s) identified above. Due to computation efficiency, it is impractical to compute the AIC statistics for all possible regression models. Stepwise methods such as forward selection method and backward elimination method are developed to avoid the complexity of exhausted search [9-11]. However, in the case of backward selection method, a variable once eliminated can never be reintroduced into the model, and in the case of forward selection, once included can never be removed [9, 11]. Thus, the stepwise regression method which combines forward selection method and backward elimination method was applied to compute the AIC statistics. Once the estimated regulatory parameters were examined using the AIC model selection criteria, the student's t-test was employed to calculate the *p*-values for the regulatory abilities (a_{ij}'s) under the null hypothesis $H_0 : a_{ij} = 0$ [8, 9] to determine the significant regulatory interactions. The *p*-values computed were then adjusted by Bonferroni correction to avoid a lot of spurious positives [8, 9]. The interactions which adjusted *p*-value $\leqq$ 0.05 were determined as significant interactions and be preserved in the gene regulatory network.

Statistical measurements of the performance

Sensitivity and specificity are statistical measures of the performance of screening /diagnostic test. The sensitivity measures the proportion of true positives that are correctly identified by the test and the specificity measures the proportion of true negatives that are correctly identified by the test [9, 12]. More specifically, the sensitivity and the specificity are defined as the following equations.

$$\text{sensitivity}=\frac{\text{number of True Positives}}{\text{number of True Positives}+\text{number of False Negatives}} \qquad \text{(S5)}$$

$$\text{specificity}=\frac{\text{number of True Negatives}}{\text{number of True Negatives}+\text{number of False Positives}} \qquad \text{(S6)}$$

In a diagnostic test, for example, a sensitivity of 100% means that the test recognizes all sick people as such. Thus, in a high sensitivity test, a negative test result is used to rule out the disease. A specificity of 100% means that the test recognizes all healthy people as healthy. Thus a positive result in a high specificity test is used to confirm the disease.

Further, positive predictive value measures the proportion of objects with positive test results which are correctly diagnosed/screened. Negative predictive value measures the proportion of objects with negative test results which are correctly diagnosed/screened [13]. More specifically, the positive predictive value and the negative predictive value are defined as the following equations.

$$\text{positive predictive value}=\frac{\text{number of True Positives}}{\text{number of True Positives}+\text{number of False Positives}} \qquad \text{(S7)}$$

$$\text{negative predictive value}=\frac{\text{number of True Negatives}}{\text{number of True Negatives}+\text{number of False Negatives}} \qquad \text{(S8)}$$

In a diagnostic test, a high positive predictive value means that a positive test result will have a high chance of identifying an individual with the disease. A high negative

positive value indicates that a negative test result has a high chance of identifying an individual who does not have the disease.

References

1. Bar-Joseph Z, Gerber G, Gifford DK, Jaakkola TS, Simon I: **A new approach to analyzing gene expression time series data.** In *Proceedings of the sixth annual international conference on Computational biology*. pp. 39-48. Washington, DC, USA: ACM; 2002:39-48.
2. De Boor C: *A practical guide to splines : with 32 figures.* Rev. edn. New York: Springer; 2001.
3. Faires JD, Burden RL: *Numerical methods.* 2nd edn. Pacific Grove, CA: Brooks/Cole Pub. Co.; 1998.
4. Coleman TF, Hulbert LA: **A direct active set algorithm for large sparse quadratic programs with simple bounds.** *Mathematical Programming* 1989, **45:**373-406.
5. Gill PE, Murray W, Wright MH: *Practical optimization.* London ; New York: Academic Press; 1981.
6. Akaike H: **A new look at the statistical model identification.** *Automatic Control, IEEE Transactions on* 1974, **19:**716-723.
7. Johansson R: *System modeling and identification* Englewood Cliffs, NJ Prentice-Hall; 1993.
8. Mendenhall W, Sincich T: *Statistics for engineering and the sciences.* 4th edn. Upper Saddle River, N.J.: Prentice-Hall; 1995.
9. Pagano M, Gauvreau K: *Principles of biostatistics.* 2nd edn. Pacific Grove, CA: Duxbury; 2000.
10. Hocking RR: **A Biometrics invited paper. The analysis and selection of variables in linear regression.** *Biometrics* 1976, **32:**1-49.
11. Seber GAF, Lee AJ: *Linear regression analysis.* 2nd edn. Hoboken, N.J.: Wiley-Interscience; 2003.
12. Altman DG, Bland JM: **Statistics Notes: Diagnostic tests 1: sensitivity and specificity.** *BMJ* 1994, **308:**1552.
13. Altman DG, Bland JM: **Statistics Notes: Diagnostic tests 2: predictive values.** *BMJ* 1994, **309:**102.
