# Supplementary material for: Global screening of potential Candida albicans biofilm-related transcription factors via network comparison
Source: BMC Bioinformatics. 2010 Jan 26;11:53. doi: 10.1186/1471-2105-11-53 (PMC2842261; doi:10.1186/1471-2105-11-53)
Supplement: Additional file 4 — Supplementary Figure S1. Supplementary figure S1 shows the schematic view of the biofilm regulatory network. [file 1471-2105-11-53-S4.PDF]

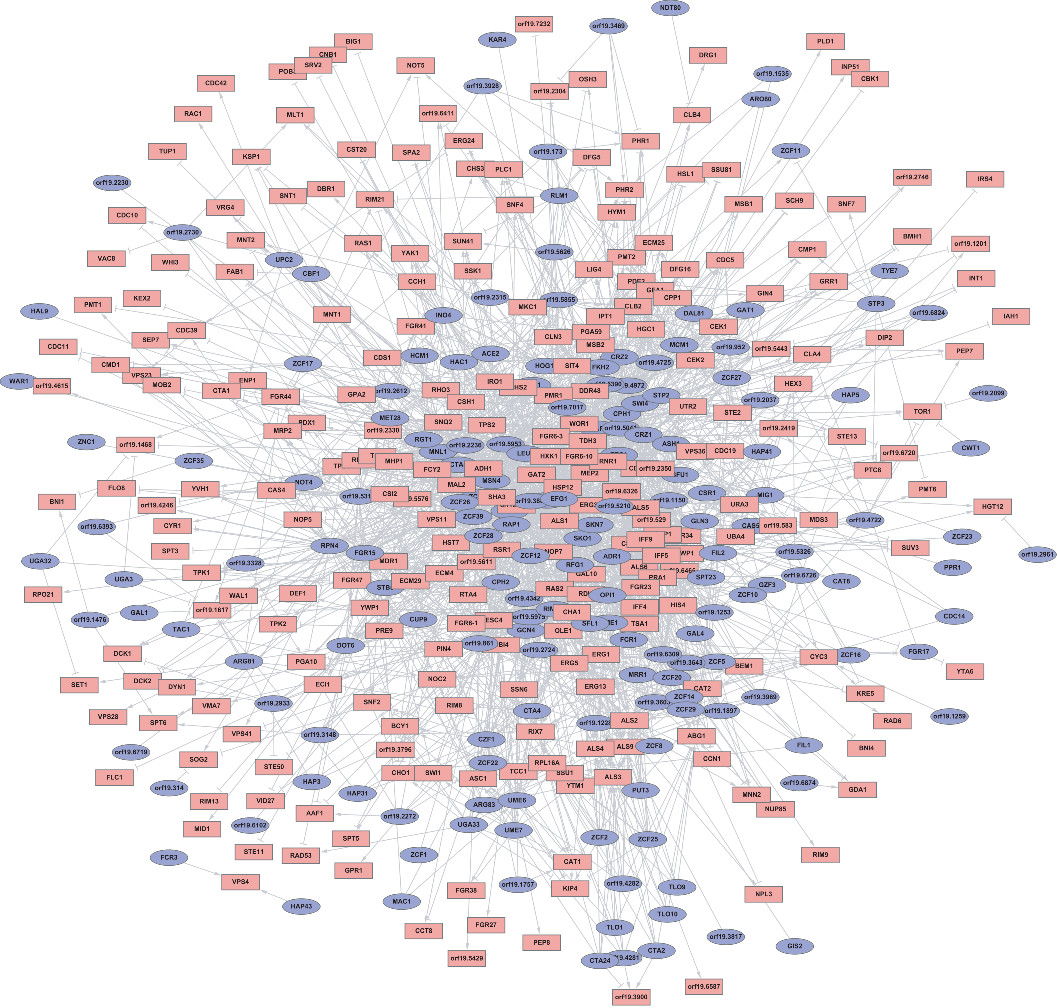

Supplementary Figure S1 - The biofilm gene regulatory network.The figure was plotted using Cytoscape.
