# Supplementary figures and images for: Global screening of potential Candida albicans biofilm-related transcription factors via network comparison
Source: BMC Bioinformatics. 2010 Jan 26;11:53. doi: 10.1186/1471-2105-11-53 (PMC2842261; doi:10.1186/1471-2105-11-53)

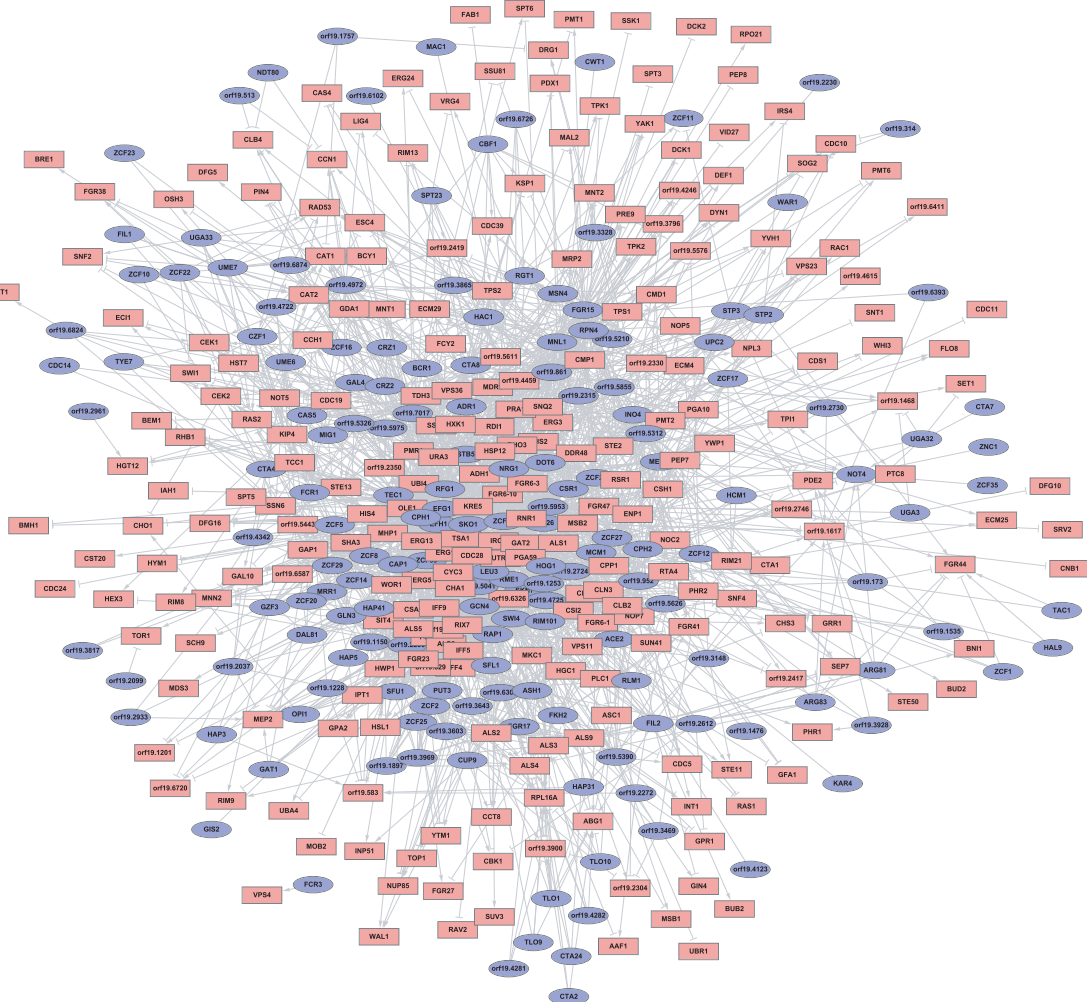

Supplement: Additional file 5 — Supplementary Figure S2. Supplementary figure S2 displays the schematic view of the planktonic regulatory network. [file 1471-2105-11-53-S5.PDF]
