# Supplementary material for: Global screening of potential Candida albicans biofilm-related transcription factors via network comparison
Source: BMC Bioinformatics. 2010 Jan 26;11:53. doi: 10.1186/1471-2105-11-53 (PMC2842261; doi:10.1186/1471-2105-11-53)
Supplement: Additional file 7 — Supplementary Figure S3. Supplementary figure S3 illustrates the schematic view of the gain-of-function subnetwork. [file 1471-2105-11-53-S7.PDF]

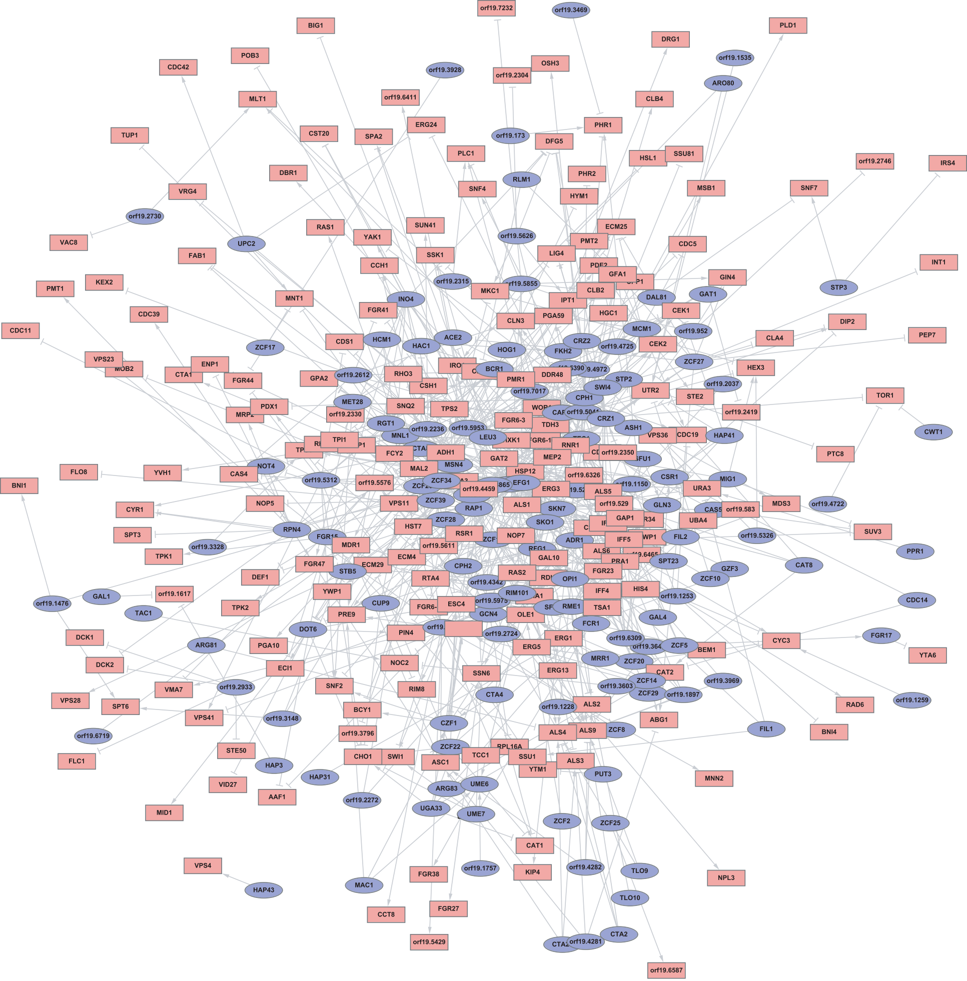

Supplementary Figure S3 - The gain-of-function subnetwork.The figure was plotted using Cytoscape.
